# Unraveling the Crimson puzzle: Two case reports/case series of hemorrhagic cystitis after combination chemotherapy with docetaxel, carboplatin, trastuzumab and pertuzumab in breast cancer

**DOI:** 10.1097/MD.0000000000041906

**Published:** 2025-03-28

**Authors:** Juan C. Ramirez, Mitchel E. Lacey, Maya S. Gowda, Grace Wang, Yolcar Chamorro, Ana C. Sandoval-Leon

**Affiliations:** a Department of Medical Education, Florida International University Herbert Wertheim College of Medicine, Miami, FL; b College of Agriculture and Life Sciences, Cornell University, Ithaca, NY; c Department of Medical Oncology, Baptist Health South Florida, Miami Cancer Institute, Miami, FL.

**Keywords:** case report, cystitis, docetaxel, dysuria, hematuria

## Abstract

**Rationale:**

Hemorrhagic cystitis can be a serious side effect of some chemotherapy drugs like cyclophosphamide and ifosfamide. Docetaxel is a taxane that is used to treat several malignancies including breast and prostate cancer. Hemorrhagic cystitis has not been described as a complication of combination chemotherapy with docetaxel, particularly docetaxel with carboplatin, trastuzumab and pertuzumab (TCHP) in breast cancer patients.

**Patient concerns:**

Both patients had a history of locally advanced human epidermal growth factor receptor 2 positive breast cancer and complaint of hematuria after their first treatment with TCHP.

**Diagnoses:**

They were diagnosed with hemorrhagic cystitis.

**Interventions:**

Docetaxel was discontinued and changed to paclitaxel.

**Outcomes:**

Both patients completed neoadjuvant chemotherapy and did not have further episodes of hemorrhagic cystitis.

**Lessons:**

Hemorrhagic cystitis is a rare complication of combination chemotherapy with docetaxel (TCHP). Clinicians should be vigilant for signs and symptoms of hemorrhagic cystitis in patients receiving docetaxel and alternative treatment option should be considered.

## 1. Introduction

Hemorrhagic cystitis (HC) manifests as a challenging clinical condition characterized by inflammation and bleeding of the bladder urothelium, stemming from either infectious or noninfectious causes.^[1–3]^ Signs and symptoms of HC can include hematuria, dysuria, or urinary frequency.^[1–3]^ Differential diagnosis includes infections, radiation therapy, chemical exposure, nephropathy, and malignancy.^[1–3]^ HC can range from mild self-resolving cases to severe, life-threatening hemorrhages requiring urgent medical interventions.^[1–3]^ While bacterial infections are the usual culprits, chronic and recurrent HC can arise from anticancer therapies like chemotherapy or pelvic radiotherapy, posing challenges for physicians and significant morbidity risks for patients.^[1–3]^ Moreover, HC can be a particular serious complication observed in certain cancer patients undergoing specific treatment, primarily associated with oxazaphosphorine alkylating agents like cyclophosphamide and ifosfamide, which generate the urotoxic metabolite acrolein which concentrates in the bladder.^[1,3–5]^ Acrolein causes increased oxidative stress and a release of inflammatory mediators, resulting in mucosal edema, hemorrhage, and cell death.^[1,3–5]^ Furthermore, HC has also been reported following administrations of dacarbazine, temozolomide, and pelvic radiotherapy, affecting approximately 6% of patients on alkylating agents.^[1,3–5]^ However, HC has not been described in patients with breast cancer receiving combination chemotherapy with docetaxel, specifically docetaxel, carboplatin, trastuzumab and pertuzumab (TCHP). Docetaxel is a semi-synthetic taxane used in treating various cancers such as breast, lung, gastric, head and neck, and prostate cancers that does not typically cause hemorrhagic cystitis.^[5]^

Docetaxel is a chemotherapeutic agent used in the management of several malignancies. It is a cytotoxic plant alkaloid and a taxane derivative.^[5–8]^ Docetaxel acts by inhibiting microtubule formation and is metabolized primarily in the liver to cyclized oxazolidinedione (M4).^[5–8]^ Approximately 75% of docetaxel is excreted via bile and fecal routes and less than 10% is excreted via the renal system.^[5–8]^ Common adverse effects associated with the use of docetaxel include myelosuppression (anemia, leukopenia, or thrombocytopenia), gastrointestinal toxicity, and peripheral neuropathy.^[5–8]^ Docetaxel dose reductions are recommended in patients with hepatic impairment.^[5–8]^ Nevertheless, there are no specific dose adjustment recommendations in patients with renal impairment.^[5–8]^

Herein, we report the first 2 case reports, to our knowledge, of hemorrhagic cystitis secondary to TCHP in breast cancer patients and we hypothesize that docetaxel was the cause.

## 2. Case report

### 2.1. Case 1

**Figure 1.**
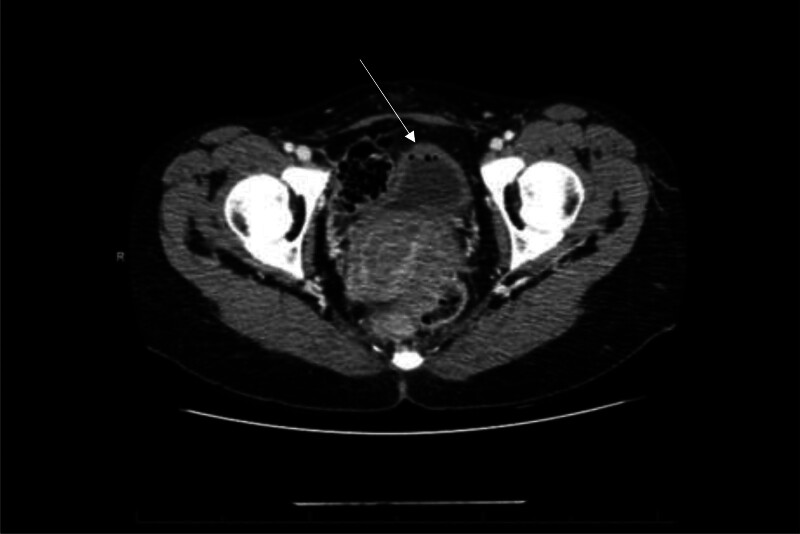
Computed tomography urogram of Case 1 showing questionable degree of minimal bladder wall thickening (white arrow).

A 43-year-old premenopausal woman with no past medical history was diagnosed with a palpable T3N1 estrogen receptor negative, progesterone receptor negative, human epidermal receptor positive left breast cancer. She started neoadjuvant treatment with docetaxel, carboplatin, trastuzumab, and pertuzumab (TCHP) given every 3 weeks with the plan to complete 6 cycles. Her regimen consisted of docetaxel 75 mg/m^2^ in combination with carboplatin area under the curve (AUC) of 6, trastuzumab loading dose of 8 mg/kg followed by 6 mg/kg and pertuzumab loading dose of 840 mg followed by 420 mg, all given every 3 weeks. Premedications included: aprepitant 130 mg, dexamethasone 12 mg and palonsetron 0.25 mg. She also received pegfilgrastim 6 mg the day after chemotherapy. Two days after the first chemotherapy infusion she started having suprapubic pain and blood in the urine with clots. For this reason, she was admitted to the hospital. Her completed blood cell count showed a normal hemoglobin (12.2 g/dL) and platelets (153 K/µL), elevated white blood cell count (WBC) of 31.84 K/µl; likely due to recent growth factor support (pegfilgrastim). Her urinalysis was positive for blood, red blood cells and WBCs. Renal function was normal with a creatinine of 0.61 mg/dL. The prothrombin time and partial thromboplastin time were normal. Computer tomography urogram showed normal kidneys and questionable degree of minimal bladder wall thickening (Fig. 1). She received intravenous fluids and was treated with empiric antibiotics for a suspected urinary tract infection. Urine culture was negative. She was evaluated by urology and the initial thought was that the hematuria could be secondary to pegfilgrastim that has been associated with hematuria.

**Figure 2.**
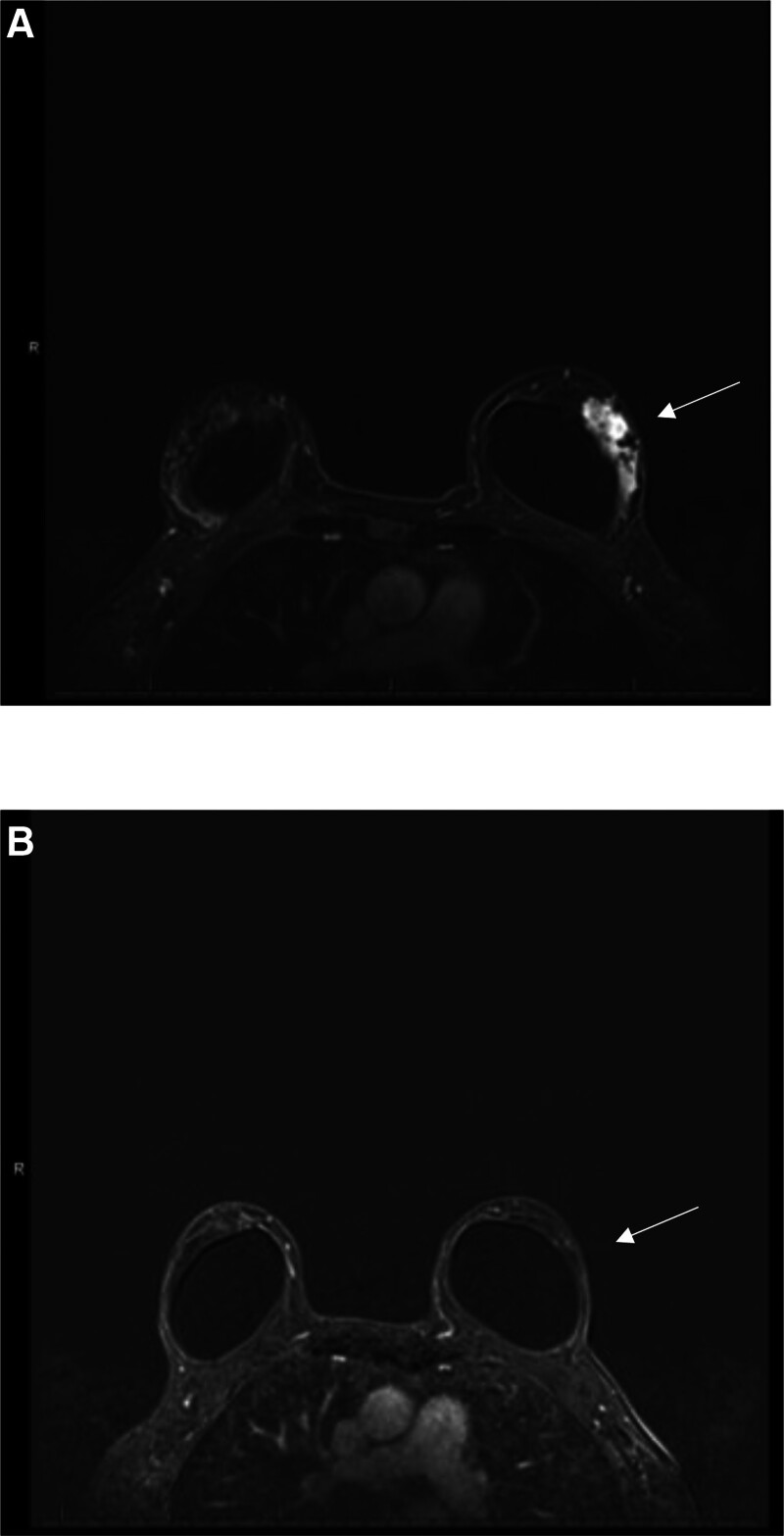
Axial T1-weighted post-gadolinium breast magnetic resonance imaging (MRI) of Case 1. (A) MRI before treatment showing an irregular enhancing mass (arrow); (B) MRI after treatment showing no pathologic enhancement (arrow). MRI = magnetic resonance imaging.

The hematuria resolved after a few days. She received her second cycle of chemotherapy without pegfilgrastim and she developed another episode of hematuria for a few days that resolved with conservative management. For the third cycle of chemotherapy docetaxel was changed to weekly paclitaxel (80 mg/m^2^), carboplatin was also given weekly with AUC of 2, both trastuzumab and pertuzumab were given every 3 weeks with no change in the dose. Premedications included: aprepitant 130 mg, dexamethasone 12 mg, diphenhydramine 25 mg, famotidine 20 mg, palonosetron 0.25 mg. No growth factor support was given. The decision to change the taxane was based on a case report of hematuria secondary to docetaxel in a patient with prostate cancer.^[5]^ She did not have further episodes of hematuria. She completed 6 cycles of neoadjuvant chemotherapy with great clinical and radiological response (Fig. 2). She had a mastectomy and sentinel lymph node biopsy showing a complete pathological response. She then completed adjuvant radiation therapy to the left breast with a total of 50.4 Gray in 28 fractions within 37 days and adjuvant trastuzumab 6 mg/kg and pertuzumab 420 mg every 3 weeks for 12 additional cycles. She has been in surveillance without evidence of recurrence of her breast cancer. She has not had further episodes of hematuria for 2 years.

### 2.2. Case 2

**Figure 3.**
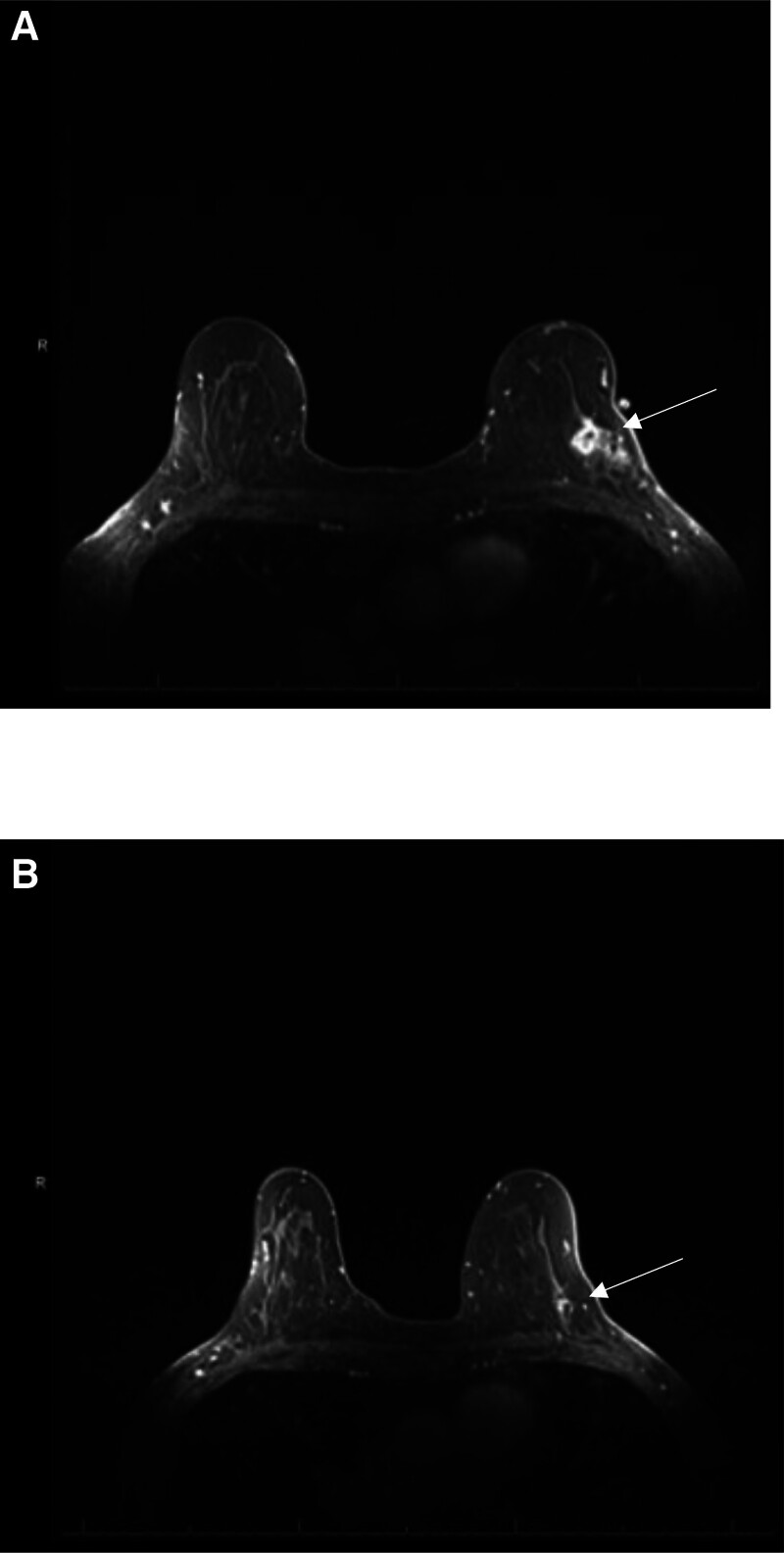
Axial T1-weighted post-gadolinium breast magnetic resonance imaging (MRI) of Case 2. (A) MRI before treatment showing a lobulated enhancing mass (arrow); (B) MRI after treatment showing resolution of the enhancing mass (arrow). MRI = magnetic resonance imaging.

A 53-year-old woman with no significant past medical history was diagnosed with a palpable T2N1 ER+, PR+, HER2+ invasive ductal carcinoma of the left breast. She started neoadjuvant systemic therapy with TCHP with the same dose and premedications as Case 1. Two days later she went to the emergency department due to blood in her urine and discomfort in the left side of her abdomen. She was hemodynamically stable. Urinalysis showed a large amount of blood in the urine and proteinuria. Her completed blood cell count was normal with a hemoglobin of 13.7 g/dL, platelets of 268 K/µL and WBC of 8.8 K/µL. The complete metabolic profile was normal including kidney (creatinine 0.58 mg/dL) and liver function tests. She had PT/INR and partial thromboplastin time done prior to starting chemotherapy and they were also normal. Urine culture showed mixed genital flora isolate described as superficial bacteria that were not indicative of a urinary tract infection. The patient was diagnosed with acute cystitis and was prescribed nitrofurantoin. The hematuria resolved after a few days. Docetaxel was changed to paclitaxel 175 mg/m^2^ for her second cycle that was given in combination with carboplatin AUC6, trastuzumab 6 mg/kg and pertuzumab 420 mg every 3 weeks for 5 additional cycles. Premedications included: aprepitant 130 mg, dexamethasone 12 mg, diphenhydramine 25 mg, famotidine 20 mg and palonsetron 0.25 mg. She did not have further episodes of hematuria. She was able to complete neoadjuvant chemotherapy with great clinical and radiological response (Fig. 3). She had a lumpectomy and sentinel lymph node biopsy showing a residual 0.4 cm tumor. The patient completed adjuvant radiation therapy to the left breast with a total of 52 Gy in 20 fractions over 34 days. She then completed adjuvant trastuzumab emtansine 3.6 mg/kg given every 3 weeks for 12 cycles and started anastrozole 1mg daily. She has been in surveillance for 2 years with no signs of malignancy and no further episodes of hematuria.

## 3. Discussion

The combination of docetaxel with carboplatin, trastuzumab, and pertuzumab (TCHP) is one of the most frequently used neoadjuvant regimens for patients with HER2+ breast cancer who are candidates for neoadjuvant chemotherapy.^[9]^ Patients undergoing neoadjuvant chemotherapy with TCHP are administered prophylactic growth factor support with pegfilgrastim to mitigate the risk of febrile neutropenia. One recognized side effect of pegfilgrastim is nephrotoxicity, manifesting as glomerulonephritis that can present with microscopic or macroscopic hematuria.^[10]^

Initially, glomerulonephritis was considered in the differential diagnosis for hematuria in the first case. But even after discontinuing pegfilgrastim, the hematuria persisted. It’s important to note that pegfilgrastim-associated nephrotoxicity typically presents with glomerulonephritis rather than hemorrhagic cystitis, indicating a distinct clinical presentation.^[10]^ Instances of painless hematuria in patients undergoing chemotherapy necessitate prompt investigation to determine whether this signals a secondary malignancy or an adverse reaction to the chemotherapy regimen.

Hemorrhagic cystitis arises from a complex inflammatory cascade involving kinins, cyclooxygenases, interleukins, and reactive oxygen species within cells.^[2–5]^ The metabolite acrolein, generated from oxazaphosphorins, is known to initiate this inflammatory process.^[2–5]^ In the context of combination chemotherapy with docetaxel, it could be possible that an unidentified metabolite could potentially trigger a similar reaction. Additionally, interactions between docetaxel and other components of the TCHP regimen may contribute to this effect.^[2–5,7,8]^ Considering that HC associated with docetaxel has been previously reported in a case involving a patient with prostate cancer and that switching from docetaxel to paclitaxel resolved the hematuria, we believe that docetaxel was likely the culprit in our 2 patients.^[5]^

Given the potential for morbidity, timely recognition and cessation of docetaxel treatment is crucial. Clinicians must remain vigilant for signs of hemorrhagic cystitis in patients undergoing combination chemotherapy with docetaxel, and alternative treatment options should be considered as appropriate. Continued research is essential to deepen our understanding of the mechanisms involved in drug-induced hemorrhagic cystitis and to develop effective strategies for its prevention and management. Both patients were able to complete neoadjuvant chemotherapy with an excellent response despite the change of taxane after the development of hemorrhagic cystitis.

## 4. Conclusion

To our knowledge, these are the first 2 cases documenting hemorrhagic cystitis secondary to TCHP in breast cancer patients, which subsequently resolved upon switching docetaxel to paclitaxel. Clinicians should be aware of this potential side effect associated with TCHP. Further studies are necessary to elucidate its underlying mechanism.

## Author contributions

**Conceptualization:** Ana C. Sandoval-Leon.

**Writing – original draft:** Juan C. Ramirez, Mitchel E. Lacey, Grace Wang, Yolcar Chamorro, Ana C. Sandoval-Leon.

**Writing – review & editing:** Juan C. Ramirez, E. Lacey, Maya S. Gowda, Grace Wang, Yolcar Chamorro, Ana C. Sandoval-Leon.
